# Attitudes, barriers, and enablers towards conducting primary care research in Banda Aceh, Indonesia: a qualitative research study

**DOI:** 10.1186/s12930-018-0045-y

**Published:** 2018-07-27

**Authors:** Ichsan Ichsan, Nur Wahyuniati, Ryan McKee, Louella Lobo, Karla Lancaster, Lynda Redwood-Campbell

**Affiliations:** 1 Medical Research Unit, Faculty of Medicine, Syiah Kuala University, Jl. T. Tanoeh Abe, Darussalam, Banda Aceh, 23111 Indonesia; 2 Department of Family Medicine, McMaster University, 100 Main St W, Hamilton, ON L8P 1H6 Canada

**Keywords:** Research, Primary health care, Physicians, Primary care, Qualitative research, Asia, southeast, Indonesia, Developing countries

## Abstract

**Background:**

Conducting university-based research is important for informing primary care, especially in lower- and middle- income countries (LMICs) such as Indonesia. Syiah Kuala University (SKU), the largest educational institution in Aceh province, Indonesia, is actively establishing itself as a leader in research innovation; however, this effort has not yet demonstrated optimum results. Understanding faculty members’ perceptions of how research is conducted in this setting is crucial for the design and implementation of successful and sustainable research strategies to increase the quantity and quality of primary care research conducted at LMIC universities. The objective of this study was to identify current attitudes, barriers and enablers/facilitators towards primary care research participation and implementation in this higher education institution.

**Methods:**

A descriptive-interpretive qualitative study was conducted. 29 participants, representing 90% of all faculty members providing primary care, were included. A mixed-methods approach was used, combining the use of a participant survey with 10 focus group discussions. Participants were encouraged to complete the survey in either English or Bahasa Indonesia. All of the focus group discussions were recorded, transcribed and translated into English. Thematic content analysis of these transcripts was carried out.

**Results:**

The majority of participants agreed that SKU has set research as a priority, as it is one of the three pillars of higher education, mandatory in all Indonesian higher education institutions. This research identified many barriers in conducting research, i.e. weak research policy, lack of research funding and infrastructure, complicated research bureaucracy and administrative process, as well as time constraints for conducting research relative to other duties. Participants expressed that personal motivation was a very important enabler/facilitator for increasing research activities. In order to improve research productivity, the majority of participants suggested that having local awards and formal recognition, having the opportunity to partner with local business and communities, provision of incentives, and having access to a research help-desk would be beneficial.

**Conclusions:**

Generally, participants showed a supportive and positive attitude towards research, and provided examples of how to improve research productivity in the Asian university context.

## Background

Research capacity has long been the hallmark of educational institutions. In medicine, the quality of care provided to patients is often dictated by the ability of physicians to apply high-quality research to their clinical practice. Research in primary care largely aims to seek a better understanding of disease management in relation to the individuals, families and the community, and to evaluate the effectiveness and efficiency of healthcare practices and health policies. Continuous advances in disease prevention and management through relevant research creates the foundation for a dynamic healthcare system that can soundly address the needs of a community. In many lower- and middle-income countries (LMICs), tremendous strain is placed on health care systems by both the increasing burden of chronic, non-communicable disease and high rates of infectious disease [1–7]. Prevalence of chronic and infectious disease may be combated and controlled by increased research capacity, and by improving the evidence base used to inform clinical practice [8, 9]. However, unlike high-income countries, many LMICs lack the human resources and financial resources to sustain high-level research [1, 8, 10]. Furthermore, LMICs have unique clinical contexts and research needs that are often not adequately addressed by research carried out in North America and Europe. Of the approximately US$ 240 billion dollars spent worldwide on health research each year (2010 data), approximately 90% is used to fund research on health conditions that affect approximately 10% of the global population [11, 12].

Nevertheless, there is an increasing demand for the use of evidence-based practice around the world [13, 14]. Countries in the Asia Pacific region, such as Indonesia, have recently seen an increase in both support for and interest in evidence-based medical research, and are becoming increasingly engaged in clinical epidemiology and clinical research [9, 15–17]. However, challenges to research practice and implementation still exist [9].

In order to increase sustainable research capacity in developing countries such as Indonesia, it is important to understand the context of the local health research landscape, and to gain insight into factors that influence clinical research both positively and negatively.

### Literature review

There is a paucity of research exploring the various barriers and facilitators to implementation of health research in Indonesia, especially with respect to the views of clinicians and university faculty members. There is, however, a small amount of literature describing the research output from Indonesia, and a moderate amount of literature exploring the knowledge and attitudes of medical students towards research practice in LMICs. Additionally, one paper was identified that explored the perspectives of junior faculty members of Pakistani medical schools on the topic of health research.

### State of health research in Indonesia

The World Health Organization (WHO) estimates that countries in the South East Asia Region (SEAR), including Indonesia, bear approximately 30% of global disease burden [18]. Despite this, Indonesia spends approximately 2.2% of its gross domestic product (GDP) on health expenditures, the lowest of all SEAR countries [5]. Between 2000 and 2010, approximately 5.0% of peer-reviewed epidemiological publications from the SEAR region were from Indonesia, a lower proportion than other countries in the area [5]. Data from the University of Indonesia shows that approximately 1168 research studies were conducted between 2013 and 2017, of which only about 64 (39%) have had their results published [19, 20]. There appears to be a disparity between the actual disease burden in Indonesia and the amount of health expenditure and epidemiological research output [5]. Moreover, the 2014 literature review by Widyahening et al. Estimated that only 21% of published articles from Indonesia between 2008 and 2010 were of high reporting quality, and only 15% were of high methodological quality [9]. Several barriers to performing clinical epidemiology and evidence-based medicine have been identified: underutilization of data for planning; need for capacity building; linking of evidence to practice and policy; developing collaborative research networks (both nationally and internationally); and need for improved infrastructure, funding and technical expertise [15, 21].

### Perspectives of medical students and faculty members on health research

The majority of research on this area has been conducted in South Asia, Northern Africa, and the Middle East. Studies from India (n = 2), Iran (n = 2), Pakistan (n = 3), Sudan (n = 1) and Saudi Arabia (n = 2), as well as a multicentre study across three Middle Eastern countries (Saudi Arabia, Bahrain, and Kuwait), were identified. Generalizability of results to SEAR countries such as Indonesia may be limited, however, a number of consistent barriers and facilitators to health research were identified.

Overall, medical students in LMICs were found to have a favourable view of research, with 82–97% of surveyed students indicating that research was important to the practice of medicine [22–26], and 64–91% agreeing that conducting research in medical school is important and should be included in the curriculum [22–28]. When medical students were asked about their participation in research, the percentage of students who had experience conducting research varied greatly. Results from six studies ranged from 52 to 61% [23–28]. The study by Osman found that 94% of medical students had participated in research under a supervisor [22], and the study by Memarpur et al. found that all surveyed participants had participated in research [29]. Overall knowledge of research concepts was low to moderate [22–28], and the percentage of students who had published was also low, ranging between 7 and 34% [25, 26, 30].

Barriers to conducting and participating in research included: insufficient time for research [23, 24, 26, 28–30], lack of adequate funding and financial incentives [22, 23, 26, 28, 29, 32], lack of supervisors and mentors [22–24, 26, 28, 30], lack of training and research curriculum [23–26, 29], and lack of adequate research facilities [26, 28]. Facilitators of research amongst medical students included: formal training in research skills [24–28, 30], competent and motivated supervisors [22, 24, 29], inclusion of research in the curriculum [24, 25], and having time set aside for research activities [23].

The 2009 study by Sabzwari et al. Focused on the attitudes and experiences of junior faculty members at four medical schools and teaching hospitals. They found that 42% of surveyed participants were currently involved in clinical research. 85% of participants felt that research was helpful; however, 83% admitted that research was difficult to conduct. Barriers to participation in clinical research included: lack of training, lack of time to perform research, lack of statistical support, lack of mentorship, and lack of financial incentives. Facilitators to conducting research included prior formal training in research, having dedicated time for research activities, and availability of mentors and research support [31].

### Objectives

It is clear that there are many barriers in performing health research in LMICs, however, there is very little available literature exploring the research needs of Indonesian primary care researchers. Thus, this study sought to explore the environment and culture of health research in the Family Medicine department of a major Indonesian university. The objectives of this study were: (1) to explore current attitudes of faculty members towards primary care research, including participation and implementation; (2) to identify any perceived barriers in conducting and producing research; and (3) to identify enablers/facilitators for promoting an environment conducive to research.

## Methods

### Setting and participants

This study took place at Syiah Kuala University (SKU), located in Banda Aceh, in the province of Aceh, Indonesia, and was performed in collaboration with the Department of Family Medicine at McMaster University in Hamilton, Ontario, Canada. SKU Department of Family Medicine faculty members working as primary care physicians were invited to participate in this study. Participants were recruited using a purposive sampling method. 29 participants who consented to participate were enrolled. This number represented 90% of all faculty members providing primary care at SKU. This study was approved by the Hamilton Integrated Research Ethics Board, and the Syiah Kuala University Research Ethics Board.

### Design

This study used a mixed-methods approach, combining the use of a participant survey with focus group discussions, focusing on qualitative descriptive-interpretive analysis. Participants were encouraged to complete the survey in either English or Bahasa Indonesia. All of the focus group interviews were digitally recorded, transcribed and translated into English.

The participant survey contained 28 questions, including demographic questions (age, gender, level of training, additional degrees), questions regarding previous research experience (yes/no), questions on attitudes towards research and research skills (5 point Likert scale), and questions on perceived barriers and facilitators to conducting research (5 point Likert scale). Faculty members who agreed to complete the survey were subsequently invited by the Principal Investigator to participate in focus group discussions, which were held at the Faculty of Medicine.

The semi-structured focus group discussions utilized a mixture of both closed- and open-ended questions in order to collect demographic data, as well as eliciting faculty member perspectives on the definition and requirements of research, general attitudes towards conducting research, barriers to conducting research, and incentives and facilitators to conducting research. Ten focus group discussions (including 29 participants) were conducted. They were facilitated by a member of the research team (a lecturer at the Faculty of Medicine at SKU). Focus group interviews were audio recorded, and then transcribed and translated into English by an external translator.

### Data analysis

Participant surveys were organized using Microsoft Excel. Mean aggregate scores were calculated based on individual subject area. To simplify analysis, Likert scale values of 1 and 2 were combined to represent disagreement with the study statement, whereas scale values of 4 and 5 were combined to represent agreement with the study statement. A value of 3 was considered neutral.

Focus group interview transcripts were analyzed by five research team members using a qualitative, descriptive approach. Each transcript was coded independently to capture emerging themes and sub-themes. These were discussed, organized and consolidated by all members of the research team in a collaborative manner. Analysis focused on understanding current attitudes towards research and addressing current barriers and enablers/facilitators to conducting research. Data collection occured alongside data analysis, so as to ensure that new emerging themes were fully explored. Themes from qualitative analysis were triangulated with the survey results to ensure convergence of information and agreement between the two different modalities of data collection.

## Results

### Participant survey

This study included 29 participants, all of whom were members of the Faculty of Medicine at SKU. The majority of participants were female (65.5%), aged 27–38 years (75.9%), serving as a medical doctor (86.2%), and holding a master’s degree (79.31%). Results from the participant survey are presented in Tables 1, 2, 3, 4, 5.

**Table 1:** Demographic data of participants (faculty members), n (%)

Sex	
Male	10 (34.5)
Female	19 (65.5)
Age range	
27–38 years old	22 (75.9)
41–57 years old	7 (24.1)
Level of training	
Medical doctor	25 (86.2)
Non medical doctor	4 (6. 9)
Additional degree	
Ph.D. degree	6 (20.9)
Master degree	23 (79.3)

**Table 2:** Research experience among study participants, n (%)

Research experience (n = 29)	Yes	No
Have you conducted research before?	28 (96.6)	1 (3.4)
Are you currently involved in a research project?	16 (55.2)	13 (44.8)
Received formal training in conducting research?	25 (86.2)	4 (13.8)

**Table 3:** Research attitudes amongst study participants, n (%)

Research attitudes and skills (n = 29)	Agree	Neutral	Disagree
I am interested in conducting research	29 (100.0)	0 (0.0)	0 (0.0)
Research should be a top university priority	29 (100.0)	0 (0.0)	0 (0.0)
Research has a positive effect on SKU	29 (100.0)	0 (0.0)	0 (0.0)
Research promotes critical thinking	29 (100.0)	0 (0.0)	0 (0.0)
Research improves patient care	25 (86.2)	4 (13.8)	0 (0.0)
Research helps professional enhancement	29 (100.0)	0 (0.0)	0 (0.0)
Research helps to change health policy	28 (96.6)	1 (3.4)	0 (0.0)
There should be dedicated time allotted for research	27 (93.1)	1 (3.4)	1 (3.4)
I am confident in my abilities to conduct research	27 (93.1)	2 (6.9)	0 (0.0)

**Table 4:** Enablers/facilitators to conducting research, n (%)

Enablers/facilitators to conducting research (n = 29)	Agree	Neutral	Disagree
Career advancement	27 (93.1)	1 (3.4)	1 (3.4)
Support from administrators	18 (62.1)	9 (31.0)	2 (6.9)
Pursuit of personal interest	18 (62.1)	11 (37.9)	0 (0.0)
Pursuit of further education	26 (89.7)	3 (10.3)	0 (0.0)
Release time from teaching duties	11 (37.9)	10 (34.5)	8 (27.6)
Opportunity to involve students	26 (89.7)	2 (6.9)	1 (3.4)
Opportunity to work with business and community partners	27 (93.1)	2 (6.9)	0 (0.0)
Formal recognition by university	27 (93.1)	2 (6.9)	0 (0.0)
Other enablers/facilitators	There should be allocated time for three pillars of higher education: research, education, community services		
	Lecturer’s performance appraisal and career rank system		
	Establishment of research networks, both within the university and with other institutions		
	Personal interest

**Table 5:** Barriers to conducting research, n (%)

Barriers to conducting research (n = 29)	Agree	Neutral	Disagree
Lack of research experience	20 (69.0)	2 (6.9)	7 (24.1)
Lack of training	23 (79.3)	2 (6.9)	4 (13.8)
Lack of release time by university	15 (51.7)	6 (20.7)	8 (27.6)
Lack of administrative support	19 (65.5)	4 (13.8)	6 (20.7)
Lack of infrastructure	26 (89.7)	2 (6.9)	1 (3.4)
Lack of grants/bursaries available	15 (51.7)	7 (24.1)	7 (24.1)
Lack of recognition by university	7 (24.1)	9 (31.0)	13 (44.8)
Lack of financial incentives	23 (79.3)	3 (10.3)	3 (10.3)
Other barriers identified	Personal time management		
	Personal motivation		
	Research funds should be allocated per faculty to reduce the bureaucratic procedures for obtaining funds		
	Complicated procedures for submitting final research reports to the university		
	Lack of mutual support and self-motivation		
	Inadequate laboratory facilities and relatively limited research grants		
	Lack of rewards		
	Division of research groups, lack of research on specific and sustainable topics		

### Focus groups

#### Attitudes

There was a consensus amongst the respondents that SKU had already set research as a high priority. In Indonesia, research is considered one of three mandatory pillars of higher educational programs, along with education and community service.

“*We have like 3 pillars, one is the research obligation” (FGD3, 83*–*4)*

*“(research serves to) increase my personal or institutional pride (FGD2, 45*–*6)*

*“For me it is a passion for research, and it is also our duty as a faculty member”* (*FGD5, 142*–*43*)

This institution has also established a research master plan; however, results from this study found that this plan is not supported by an effective research policy, especially at the faculty and study program levels. For example, participants identified a lack of research administrative support at the faculty level, which has lowered faculty member’s interest in conducting primary care research.

In Indonesia, all higher education institutions are ranked by a third-party agency according to three possible levels of accreditation, with A-level being the highest. All participants agreed that SKU provides many research opportunities, especially since 2015 when it obtained A-level accreditation. These opportunities arise from good working relationships between the university and various national and international institutions; an increasing number of international meetings, seminars, and conferences being held on-site; high rates of tropical diseases; local traditions and natural resources; as well as strong communication between the university and the government. In contrast, many of the research grants provided by local, national, and international institutions are only available for applicants with PhD or Master’s degrees, which participants felt created obstacles for beginner researchers in certain circumstances.

#### Enablers/facilitators

The majority of participants felt that personal motivation for conducting research was one of the most important aspects for increasing the number of research activities. In order to improve research productivity, the participants suggested that the university provide local awards, expand opportunities to work with local businesses and communities, institute a formal recognition initiative, provide incentives for conducting research, and implement a research help desk to support those engaging in research.

*“I think ambition or personal desire was indeed quite an important role in conducting research” (FGD5 116*–*117)*

*“Recognition will make the purpose become stronger. So the new spirit will be born” (FGD4, 179)*

Additionally, building teamwork with students was felt to be a positive factor for improving motivation for conducting research. This collaboration was found to give credence to faculty members with respect to implementing research and building capacity.

*Working with students; “It seems like the chance to facilitate students’ need makes lecturers are very enthusiastic to do research*–*than just doing it for their own” (FGD9, 188*–*189)*

#### Barriers

Participants indicated that university-provided research grants are not always distributed in an equitable manner amongst all faculties or individual faculty members; this led to a reduction in research capabilities. The availability of research grants was not thought to be equal to the faculty members’ research interests, leading to high levels of competition for access. It was also felt that these grants are not sufficient to cover all research needs, including primary care research. There was a consensus amongst the majority of participants that the university should allocate a certain amount of grant funding to each faculty to be managed independently and distributed to each department. Participants believed that this strategy may boost faculty member interest in research. Complicated research bureaucracy and financial administrative processes (including a lack of administrative support staff) was felt to be a major barrier amongst participants.

*“I believe all Universities in Indonesia provide grants for research. But here, the main problem is the complicated bureaucracy that makes the fund can’t be received as soon as possible” (FGD8, 121*–*123)*

Due to the university’s mandate of actualizing the three pillars of higher education, the majority of participants agreed that the amount of time available for conducting research is inadequate.

*“I am eager to conduct as much research as possible, but I don’t have any spare time to conduct the research.” (FGD2, 63*–*4)*

*“We don’t have any time released from university to conduct research, but the university demands as much researches as possible in a year.” (FG2 263*–*4)*

Participants perceived that most available time is spent on the educational pillar (i.e. teaching). This is made worse by a workload that is not evenly distributed amongst faculty members. Faculty members who also work as a practicing physician have an especially hard time due to the additional responsibility of patient care. It was suggested that the time allotted for implementation of each of the three pillars should be clearly regulated, especially for research. While the university has seen improvements in infrastructure over the past few years, these have not yet helped improve research infrastructure. The majority of participants agreed that there is a lack of research laboratories equipped with sufficient instruments, as well as a lack of adequate power supply to run necessary activities. The availability of skilled human resources (Master-and/or Ph.D.-level graduates both from national and international universities), while improving, still contributes to poor research infrastructure. The lack of a high-quality research infrastructure has been identified as one of the most important problems faced by faculty members, which has led many, especially those from the Faculty of Medicine, to conduct more community-based research.

*“Many of us have taken our masters…..But when we return to our faculty….still lack of those tools, we finally decide to only do the survey….So lack of infrastructure makes our talent not amplified, and our interest not distributed.” (FGD6, 75*–*80)*

*“lack of training is not a problem” (FGD8 94*–*95)*

Additionally, gender and traditional gender roles appear to be prominent obstacles to conducting research amongst women participants.

*“because when (women) get home, they instinctively change their function. They will be in housewives mode.” (FGD2, 280*–*281)*

Many female participants felt that their home and domestic duties imposed additional time constraints that further prevented them from conducting research.

## Discussion

This study provides an important overview of the attitudes towards research held by faculty members in primary care at a higher education institution in an LMIC, as well as the barriers and enablers/facilitators that influence research capacity and production. It is likely that other universities and institutions in LMICs and across the Asia Pacific region face the same challenges.

It is widely accepted in the Indonesian context that the availability of a research master plan is important for higher education institutions; however, results from this study find that this alone may not be enough to increase the culture and productivity of research. This study shows that the implementation of the three pillars of higher education is not fully facilitated by the university with regards to research obligation. Results found that a lack of research support prevents faculty members from fully engaging in research activities, findings that are supported by previous published research. This suggests that many of the barriers faced by researchers are common across various LMIC settings and contexts, including: inadequate funding and financial incentives [31, 32], lack of available time for conducting research [23, 24, 26, 28–31], poor research policy [23–26, 29], lack of adequate research infrastructure and facilities [26, 28], and a lack of financial and administrative support [31].

Nevertheless, this study also supports the overall positive attitude that researchers in LMICs tend to have towards research [22–28, 31]. The three pillars of higher education (research, education and community service), mandated as national requirements for higher education institutions, have become a unique cardinal enabler/facilitator in strengthening research capacity in the Indonesian context. This system could potentially be adapted by other LMICs as a means of increasing research capacity, especially in the Asia Pacific region.

As one of many Indonesian universities, SKU, situated in the province of Aceh, has many unique opportunities for research. Aceh is known as being a “living laboratory” for many researchers from around the world, due in part to the fact that it is a tropical region, and thus is home to many endemic tropical diseases. A rich Acehnese tradition and plentiful natural resources can be leveraged and explored by faculty members looking to expand their research practices and scope. It is likely that many other areas in the Asia Pacific region would have their own similar yet unique contexts that might make them attractive locations for prospective researchers. Additionally, the growing involvement of local faculty members in international meetings, seminars, and conferences adds positive value to research initiatives across Indonesia and the Asia Pacific region. Faculty members are often aware of these opportunities, however, they may face difficulties in the operationalizing and production of high-impact research.

On the other hand, results from this study indicate that the interest level of faculty members in conducting research is low, due to inequality of funding, and a lack of resources, infrastructure, and research support at the faculty and study program levels. In order to promote and support successful research initiatives, tools such as a comprehensive and effective research policy (available at the faculty and study program levels), are required.

This study also found that female faculty members of higher education institutions may also face additional time constraints, due in part to traditional gender roles. In general, women in many Asian countries often have additional responsibilities, both professionally and at home. As faculty members, women are required to participate in teaching, research, and community service. If they are also practicing primary care doctors, they will have additional patient care duties. Women faculty members may also have increased amounts of domestic duties outside of work relative to their male counterparts, which may decrease the amount of time they have available to conduct research. This disparity needs to be addressed, not only in Indonesia, but also in many other LMICs.

## Conclusions

This study served as a critical needs assessment that evaluates the research environment of primary care physicians at a major Asian university. By identifying current attitudes, barriers, and facilitators of conducting research, this study was able to identify a number of recommendations that may help to increase research capacity. These findings were found to support previous evidence regarding barriers to research production in LMICs. Future research will play an integral role in assessing the development of primary care in LMICs, and will ensure that healthcare development is producing measurable improvements for patients.

## Abbreviations

GDP: gross domestic product
LMIC: lower- and middle-income country
SEAR: south east Asia region
SKU: Syiah Kuala University
WHO: World Health Organization
